# Development of oligonucleotide microarrays for simultaneous multi‐species identification of *P* *hellinus* tree‐pathogenic fungi

**DOI:** 10.1111/1751-7915.12341

**Published:** 2016-02-08

**Authors:** Yuh Tzean, Po‐Yao Shu, Ruey‐Fen Liou, Shean‐Shong Tzean

**Affiliations:** ^1^ Department of Plant Pathology and MicrobiologyNational Taiwan UniversityTaipeiTaiwan

## Abstract

Polyporoid *P* *hellinus* fungi are ubiquitously present in the environment and play an important role in shaping forest ecology. Several species of *P* *hellinus* are notorious pathogens that can affect a broad variety of tree species in forest, plantation, orchard and urban habitats; however, current detection methods are overly complex and lack the sensitivity required to identify these pathogens at the species level in a timely fashion for effective infestation control. Here, we describe eight oligonucleotide microarray platforms for the simultaneous and specific detection of 17 important *P* *hellinus* species, using probes generated from the internal transcribed spacer regions unique to each species. The sensitivity, robustness and efficiency of this *P* *hellinus* microarray system was subsequently confirmed against template DNA from two key *P* *hellinus* species, as well as field samples collected from tree roots, trunks and surrounding soil. This system can provide early, specific and convenient detection of *P* *hellinus* species for forestry, arboriculture and quarantine inspection, and could potentially help to mitigate the environmental and economic impact of *P* *hellinus*‐related diseases.

## Introduction

The *Phellinus* genus *sensu lato* currently comprises 154 species (Kirk, 2014) of resupinate, sessile, polyporoid fungi, several of which are known to cause diseases such as stem rot, butt rot, root rot or tree wilt in a wide range of tree species (Van der Kamp, 1991; Castello *et al*., 1995). These tree‐pathogenic fungi include some of the most aggressive wood‐decay species identified thus far, infestations of which can devastate forest ecosystems, impact economic viability and render urban environments more vulnerable to tree hazards (Hansen and Goheen, 2000; Ann *et al*., 2002; Burdon *et al*., 2006). To date, the identification of diseased trees has primarily relied on visual inspection for signs and symptoms, pathogen isolation and characterization on selective media and other biochemical and immunological techniques (Nobles, 1965; Anselmi and Bragaloni, 1992; Jellison and Jasalavich, 2000; Clausen, 2003). However, the diagnostic process is typically laborious, time consuming and heavily reliant on experienced etiologists; moreover, the low sensitivity of such methods makes it unlikely that infestations can be detected and controlled during the relatively manageable early stages of disease (Thorn *et al*., 1996; Adair *et al*., 2002; McCartney *et al*., 2003; Luisi and Campanile, 2004). In addition, current methods are limited in their ability to identify the causative agent of *Phellinus*‐related diseases at the species level, the knowledge of which is necessary for deploying appropriate control measures (Nam *et al*., 2002) and for ascertaining whether the infestation is native or exotic in nature (Hansen and Goheen, 2000). *Phellinus* species differentiation has traditionally relied on the morphological examination of fruiting bodies, spores and basidiocarps, but these may not appear until long after infection, by which time it is often too late to save the diseased tree (Nam *et al*., 2002). Therefore, the development of accurate, fast and specific diagnostic tools that can be easily used by personnel with a minimum of training is essential for the prevention and practical management of *Phellinus* infestations.

Recent advances in molecular biology offer the possibility of alternative approaches that can efficiently identify *Phellinus* pathogens, including nucleic acid‐based techniques such as dot‐blot hybridization, restriction fragment length polymorphism analysis, single‐strand conformation polymorphism analysis and polymerase chain reaction (PCR) assays (Olive and Bean, 1999; Tsui *et al*., 2011). Polymerase chain reaction‐based methods have been used to identify *Phellinus* s.l. at a generic rank (Guglielmo *et al*., 2007; 2008), but a single assay that can efficiently pinpoint the exact disease causative agent and provide differentiation between rapid‐decaying and slow‐progressive *Phellinus* species remains elusive (Lievens and Thomma, 2005). However, methods combining nucleic acid amplification and DNA arrays have demonstrated strong potential, and their good sensitivity and specificity may allow for early and accurate detection of *Phellinus* infestation at the species level (Martin *et al*., 2000; Lévesque, 2001; Lievens and Thomma, 2005; Lievens *et al*., 2005; Tsui *et al*., 2011). In these strategies, DNA sequence samples are first amplified, then labelled with universal primers that target conserved regions flanking variable domains, and subsequently hybridized with species‐specific oligonucleotide probes on DNA arrays to screen for fungal pathogens (Saiki *et al*., 1989; McCartney *et al*., 2003; Lievens and Thomma, 2005; Lievens *et al*., 2007; 2004; Tsui *et al*., 2011). But while it is true that array technology currently offers the best chance of realizing a simple, efficient, high‐throughput pathogen detection platform that can be readily deployed in the field, one key factor has limited development thus far – a lack of discriminatory genetic regions available for species identification (Everett *et al*., 2010; Frey *et al*., 2010).

Here, we report the development of a robust high‐throughput oligonucleotide microarray system capable of simultaneously screening for multiple *Phellinus* species. The system utilizes species‐specific probes generated from internal transcribed spacer (ITS) regions, areas of non‐coding DNA located between the small subunit and large subunit ribosomal RNA (rRNA)‐coding genes, which are removed after transcription of the rRNA cistron (Lafontaine and Tollervey, 2001). It has been proposed that ITS regions can serve as a universal barcode marker for fungi, as they provide the broadest range of inter‐ and intra‐species differentiation currently known (Schoch *et al*., 2012). Internal transcribed spacer sequences have previously been used to develop primers for PCR‐based identification of *Phellinus* species (Nam *et al*., 2002; Gonthier *et al*., 2015), but to the best of our knowledge, this is the first study to utilize ITS regions in the development of DNA microarrays for detection of the *Phellinus* genus s.l. We were able to specifically resolve 17 key *Phellinus* species on our microarray system, including *Phellinus apiahynus*, *P. cesatii*, *P. gilvus*, *P. linteus*, *P. inermis*, *P. laevigatus*, *P. melleoporus*, *P. membranaceus*, *P. noxius*, *P. pini*, *P. quercinus*, *P. ribis*, *P. igniarius*, *P. formosanus*, *P. pachyphloeus*, *P. torulosus* (now reclassified as *Fuscoporia torulosa*) and *P. weirii*. Tests with template DNA sequences and field samples confirmed the sensitivity and specificity of our microarray system, which was also shown to reliably detect infections in trees before any visually identifiable symptoms of disease were observed. There is strong demand now in forestry, arboriculture and phytosanitation for pathogen detection systems that can be easily mastered and readily deployed to yield early, accurate and specific results in a very short space of time. Our *Phellinus* microarray system fulfils most of these practical requirements, and could potentially play a part in reducing the environmental and economic impact of *Phellinus* infestation worldwide.

## Results

### Design of oligonucleotide probes and DNA microarrays

**Figure 1:**
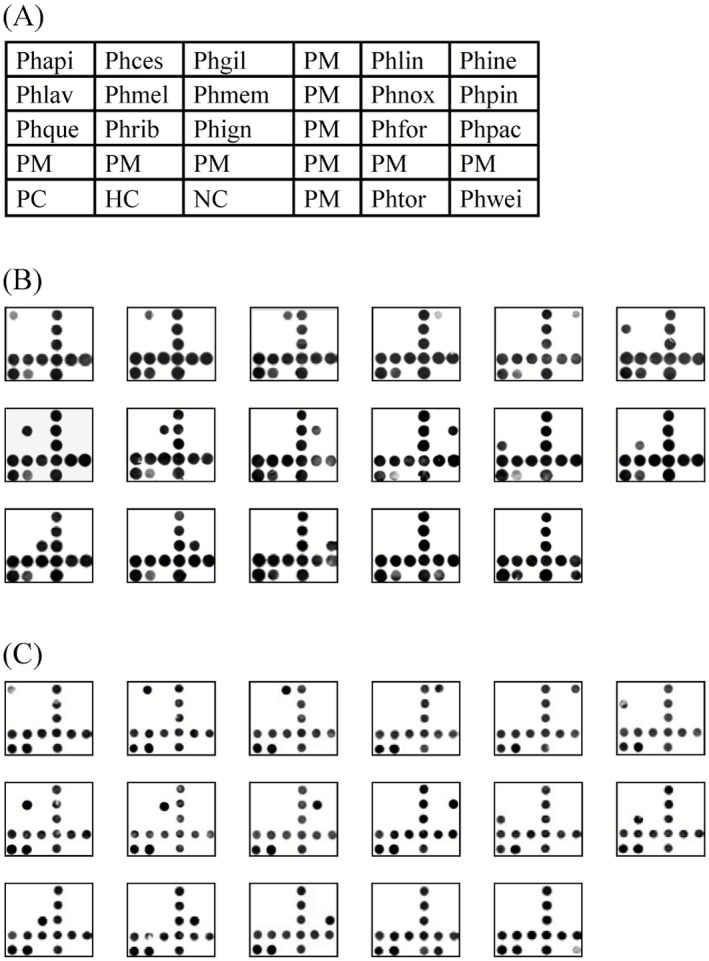
Arrangement and reverse dot‐blot hybridization results of the *P* *hellinus* oligonucleotide microarray. A. Arrangement of *P* *hellinus* species‐specific probes on microarrays. Phapi: *P* *hellinus apiahynus*; Phces: *P* *. cesatii*; Phgil: *P* *. gilvus*; Phlin: *P* *. linteus*; Phine: *P* *. inermis*; Phlav: *P* *. laevigatus*; Phmel: *P* *. melleoporus*; Phmem: *P* *. membranaceus*; Phnox: *P* *. noxius*; Phpin: *P* *. pini*; Phque: *P* *. quercinus*; Phrib: *P* *. ribis*; Phign: *P* *. igniarius*; Phfor: *P* *. formosanus*; Phpac: *P* *. pachyphloeus*; Phtor: *P* *. torulosus*; Phwei: *P* *. weirii*; PM: position marker labelled with Oligo‐(dT)10; HC: hybridization control; PC: positive control. B. Reverse dot‐blot hybridization of biotin‐primer‐labelled amplicons from target reference strains to probes on nylon membrane. C. Reverse dot‐blot hybridization of DIG‐primer‐labelled amplicons from target reference strains to probes on PVC chip.

To develop a rapid and reliable detection system capable of inter‐species differentiation, we first amplified the ITS1–5.8S–ITS2 genetic region of 17 *Phellinus* species, using universal primers previously described (White *et al*., 1990). The resulting PCR amplicons varied between 600 bp and 750 bp, and were further utilized in the design of oligonucleotide probes. The two ITS regions, ITS1 and ITS2, contain a high degree of sequence variations, which could potentially be used to generate specific probes capable of resolving different *Phellinus* species. A total of 48 probes, ranging between 28 bp and 60 bp in length and targeted to the ITS1 and ITS2 regions, were subsequently synthesized. Species‐specific sequences were designed to be located at the centre of each probe, and the probes were subjected to extensive screening with hybridization assays to confirm specificity. Through this screening process, 17 oligonucleotide probes (Table 1), one for each key *Phellinus* species targeted, were eventually selected for the development of a reverse dot‐blot hybridization DNA microarray (Fig. 1). In order to assess the applicability of the selected probes to different array systems, and to validate the reliability and efficiency of our microarrays, PCR amplicons from 27 target reference strains and 20 non‐target strains were labelled with digoxigenin‐deoxynucleoside triphosphate (DIG‐dNTP), DIG‐tagged primers, biotin‐dNTP or biotin‐tagged primers, and then reverse‐hybridized to probes spotted on either nylon membranes or PVC chips, to derive a total of eight different array platforms (Fig. 1; Fig. S1).

**Table 1:** Oligonucleotide probes used in the *P* *hellinus* microarray system

Code	Species (Reference strain)	Sequence (5' to 3')	Length	T*m*
Phapi	*P. apiahynus* (BCRC 35468)	GTCTTGTCCCCTCTTTTCATAGGAGGGGGGGGACCAGTCTTTCAAGCTGGTAT	53 bp	81.4°C
Phces	*P. cesatii* (BCRC 35431)	TAATAGTATTGTGGTGGCCATTTGCTGTTATTCATTGTTAGAAGCGGGTAACC	53 bp	76.1°C
Phgil	*P. gilvus* (BCRC 35458)	GGATTGAAAGTCGAGGCGCAAGTCTTGACTGGAGAGAAACCTTTCTACGTTTT	53 bp	79.3°C
Phlin	*P. linteus* (TFRI 1100)	AGAGTCGAAGCTGGAGTAGTCTCTGTAATCGAAACGGGCTTTTGAAGTATGCT	53 bp	77.5°C
Phine	*P. inermis* (BCRC 35430)	GTTAGTAAAAGGGGCAAGGAGTAATCCT	28 bp	58.0°C
Phlav	*P. laevigatus* (BCRC 35495)	TTGGGCGTTTAGGACGGAGTAATGAGTAGAAAGGAGGTGTAATGCTTCCATTT	53 bp	78.0°C
Phmel	*P. melleoporus* (BCRC 35429)	TCAAACTTAACTCGGTTGAAGTGGGGGGAGGAACAGTGCAAGGAGGTGGTGAA	53 bp	83.3°C
Phmem	*P. membranaceus* (BCRC 35411)	AGGTCGGTGAAAGATATAAGTGTCTCTGACGCTTGTATTGGAAGCCTTCCTAT	53 bp	76.4°C
Phnox	*P. noxius* (BCRC 35248)	CTGAAGAGAGAGAGGGAGAGGGAGAGTGGTTTATTCGTTTATTCATTTATTCG	53 bp	75.2°C
Phpin	*P. pini* (BCRC 35384)	GCCGTCGGGGTTGACTTTGTTAGTAGTGTTTCGACGCGAAAGCATACGGTCGG	53 bp	84.4°C
Phque	*P. quercinus* (BCRC 35352)	ATTGCTACAAGTATGTTAATAAGGCGAACGCACTCTTTTCGGTGTTACTAGCT	53 bp	74.6°C
Phrib	*P. ribis* (BCRC 35326)	ACGCAAGTGAGTCGTCAGTTCCCCTAAGTTGGGAGTGACTTGATTTGCTTCGT	53 bp	81.6°C
Phign	*P. igniarius* (TFRI 1543)	AGTTGGCGGTTAGTAGTCGTAAGGCGAACACTTGTCGGCGAACACTTCAATAT	53 bp	79.8°C
Phfor	*P. formosanus* (TFRI 1129)	GGGGCGAGACCTTTGAGTTCGAAGACAGTAGTTCTTTTTGCAAATGTGAGGGC	53 bp	81.5°C
Phpac	*P. pachyphloeus* (TFRI 1131)	AATCTCTGGCCATTGGTGTCTTTCATTAGACGTCGACGTGCCTTTAACTTTGA	53 bp	79.8°C
Phtor	*P. torulosus* (TFRI 1132)	CGTATGTTGGGTCGATGGAAGGTAAAGCTTTACGGCGGCATCTTCTTTAGGTC	53 bp	80.6°C
Phwei	*P. weirii* (FP 133613 (A) Sp)	GCACTTTTCGAAGTCTGTCGTCGGCTCCCATTTGGAGCAGCTGGAGGTTT	50 bp	84.0°C

a. Oligonucleotide probes were arranged on arrays as indicated in Fig. 1A.

b. Seven thymine bases were added to the 3'‐end of the probe.

b. T*m*: melting temperature.

### Reverse dot‐blot hybridization of reference strains

Reverse dot‐blot hybridization results revealed that all test isolates from target *Phellinus* species successfully hybridized with their respective oligonucleotide probes, with no cross‐hybridization observed (Fig. 1B and C; Fig. S1). No hybridization signals apart from those generated by the positive controls were observed when non‐target strains were screened. Together, these results indicate that our microarray systems were capable of achieving 100% specificity under controlled laboratory conditions. Furthermore, the use of PVC chips could potentially allow the screening process to be completed within 2 h (excluding the time required for target DNA amplication), which would represent a significant improvement over traditional *Phellinus* species identification methods that rely on morphological examination and pathogen isolation.

### Sensitivity analysis of microarrays

**Figure 2:**
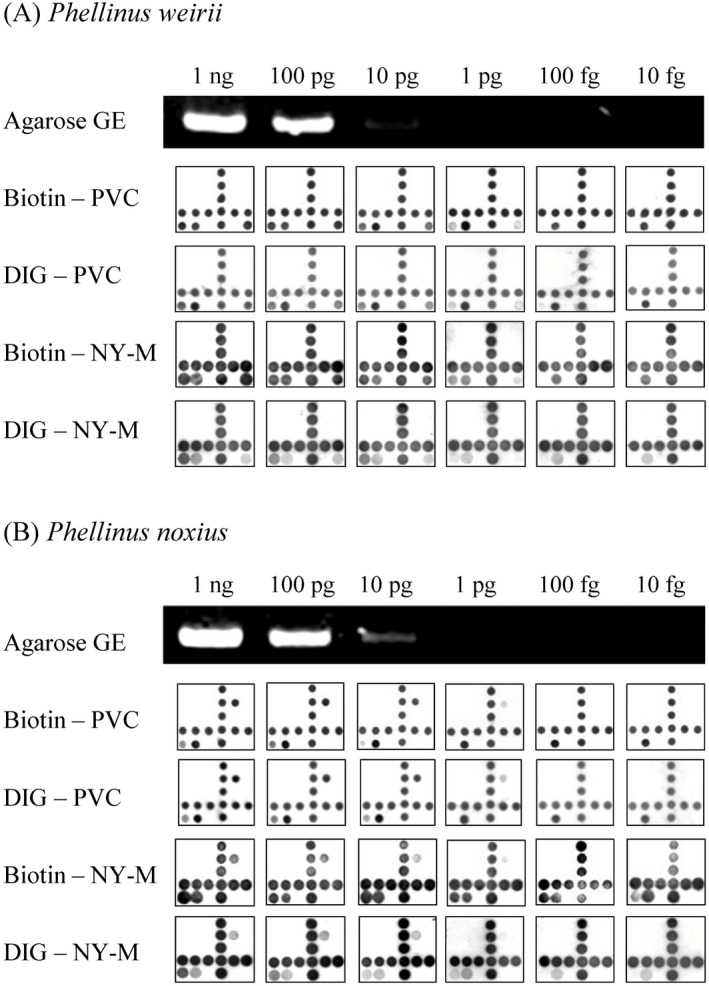
Sensitivity analysis of the *P* *hellinus* microarray. Agarose gel electrophoresis (GE) and reverse dot‐blot hybridization against microarrays were performed with PCR‐amplified (A) *P* *. weirii* or (B) *P* *. noxius* ITS primers that were serially diluted to derive samples respectively containing 1 ng, 100 pg, 10 pg, 1 pg, 100 fg or 10 fg of starting DNA. Biotin – PVC: Biotin‐labelled primer amplicons hybridized to probes on PVC chip; DIG – PVC: DIG‐labelled primer amplicons hybridized to probes on PVC chip; Biotin – NY‐M: Biotin‐labelled primer amplicons hybridized to probes on nylon membrane; DIG – NY‐M: DIG‐labelled primer amplicons hybridized to probes on nylon membrane.

To determine the sensitivity of our array system, we serially diluted template genomic DNA from *P. weirii*, a serious threat to Douglas fir viability in the Pacific Northwest of North America (Hansen and Goheen, 2000), and *P. noxius*, one of the most destructive *Phellinus* species in Taiwan (Ann *et al*., 2002). Samples respectively containing 1 ng, 100 pg, 10 pg, 1 pg, 100 fg and 10 fg of starting DNA were prepared, amplified and subjected to agarose gel electrophoresis and hybridization with different microarray systems. While DNA agarose gel bands were only visible with samples containing up to 10 pg of starting DNA (Fig. 2), our microarrays were able to accurately identify the respective *Phellinus* species with as little as 1 pg of starting DNA (Fig. 2). Biotin labelling was found to be more sensitive than DIG labelling, but no visible differences in detection sensitivity were observed between nylon membranes and PVC chips (Fig. 2).

### Analysis of complex samples and field samples

**Figure 3:**
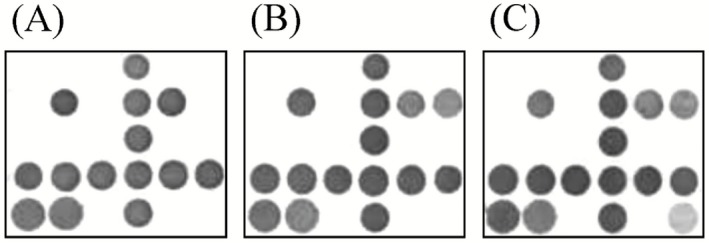
Microarray detection of complex samples within a single assay. A. Complex sample containing two *P* *hellinus* species, *P* *. melleoporus* and *P* *. noxius*. B. Complex sample containing three *P* *hellinus* species, *P* *. melleoporus*, *P* *. noxius* and *P* *. pini*. C. Complex sample containing four *P* *hellinus* species, *P* *. melleoporus*, *P* *. noxius*, *P* *. pini* and *P* *. weirii*.

In the natural environment, infestations often involve multiple fungal species, and a robust diagnostic system should have the ability to identify several different pathogenic species simultaneously. We therefore sought to assess whether our microarray system would be capable of detecting multiple *Phellinus* species within a single assay. We prepared complex samples that combined template genomic DNA from *P. noxius*, *P. melleoporus*, *P. pini and P. weirii*, and subjected the samples to reverse hybridization against our microarrays. The results showed that the arrays were capable of accurately detecting multiple *Phellinus* species in a single sample (Fig. 3).

We further prepared field samples from trees in Taiwan with suspected or confirmed *Phellinus* infestations. *Dimocarpus longan*, *Cinnamomum camphora*, *Grevillea robusta*, *Ficus microcarpa* and *Prunus campanulata* were among the tree species sampled. Our microarrays were able to detect the disease causative agent, *P. noxius* (Fig. S2), before disease symptoms were visually identifiable, and the detection results were subsequently confirmed through conventional morphological analyses and pathogen isolation methods. We further used our microarray system to assess tree seedlings from a local plant nursery for the presence of *Phellinus* infestations. Our results showed that multiple seedlings of five species (*Fraxinus formosana*, *Cinnamomum camphora*, *Koelreuteria elegans*, *Michelia champaca* and *Acacia confusa*) sampled were all free of *Phellinus* infection (Fig. S3).

## Discussion

Wood rot diseases caused by the *Phellinus* genus s.l. can induce decay in the roots, trunk and branches of almost all woody plants, and represent a serious threat to forest ecosystems and commercial arboriculture. Among the *Phellinus* species, *P. weirii* is considered to be one of the most destructive pests in the economically important Douglas fir forests of the Pacific Northwest in North America (Holah *et al*., 1993; Thies and Sturrock, Resource Bulletin PNW‐GTR‐349, 1995; Leckie *et al*., 2004); *P. noxius* has ravaged forests, plantations, orchards and urban landscapes across Japan, Taiwan and Southeast Asia (Ann *et al*., 1999; 2002; Mohd Farid *et al*., 2005; Sahashi *et al*., 2014); *P. ignarius* has been implicated as the cause of the grapevine black measles disease currently affecting vineyards in Europe and North America (Chiarappa, 1997; Gatica *et al*., 2004); and *P. pini* is viewed as one of the most dangerous invasive threats to plantation forests in Australia (Mireku and Simpson, 2002). *Phellinus*‐related diseases are transmitted when the healthy roots of susceptible trees come into contact with infected roots, stumps or soil; alternatively, *Phellinus* spores can also colonize wounds on tree trunks or branches (Leckie *et al*., 2004). In the initial stages of disease, infected trees exhibit few symptoms, and by the time crown symptoms or *Phellinus* fruiting bodies appear, a significant portion of the root system will have been destroyed, to the point that it is often too late to save the tree (Nam *et al*., 2002; Leckie *et al*., 2004). Precise identification of the exact disease‐inducing *Phellinus* species is crucial for the accurate assessment of disease progression and effective deployment of control measures, but conventional methods that rely on morphological examination and pathogen isolation are slow and inefficient (Thorn *et al*., 1996; Adair *et al*., 2002; McCartney *et al*., 2003; Luisi and Campanile, 2004). Another cause for concern involves the increasing convenience of international transport and the effects of global warming, which can facilitate the spread of *Phellinus* species to new regions or temperate habitats that were previously inaccessible due to reasons of geography or climate (Mireku and Simpson, 2002; Sahashi *et al*., 2014). Considering that *Phellinus* species have been known to survive in wood or soil for as long as 50 years (Leckie *et al*., 2004), sensitive, rapid and convenient assays that can simultaneously screen for multiple *Phellinus* species will be needed to conduct quarantine inspections of soil, seeds and saplings, timber and other wood products. In this study, we describe an oligonucleotide microarray system that combines all of these key attributes, allowing for fast, sensitive and convenient simultaneous identification of 17 key *Phellinus* species. This would potentially facilitate the early detection of disease or contamination.

Previously, microarrays have seen limited application due to the difficulty of identifying unique sequences that can be used for inter‐species differentiation (Everett *et al*., 2010; Frey *et al*., 2010), but it has been recognized in recent years that the rRNA gene regions are highly variable, and may be used to generate species‐specific markers or probes (Schoch *et al*., 2012). Nuclear large subunit (LSU) ribosomal DNA (rDNA) has been used in taxonomy to establish subdivisions within the *Inonotus* genus s.l. and *Phellinus* genus s.l. (Wagner and Fischer, 2001; 2002a,b). The ITS regions of the rRNA genes have also been used for fungal identification (Turenne *et al*., 1999; Nam *et al*., 2002; Gonthier *et al*., 2015), and it was recently proposed that ITS sequences can serve as a universal bar code marker for fungi due to their excellent inter‐ and intra‐species differentiation ability in a wide range of fungal species (Schoch *et al*., 2012). In this study, we initially designed 48 oligonucleotide probes targeting the ITS1 and ITS2 regions in 17 selected *Phellinus* species. The eventually selected probes ranged from 28–53 bp in length, excluding an additional seven thymine bases added to the 3'‐ends to improve hybridization signals (Brown and Anthony, 2000; Peplies *et al*., 2003; Leaw *et al*., 2007). It is speculated that the improved effect is due to preferential attachment of the charged support to the added thymine bases at the 3'‐end, thus leaving a greater number of oligonucleotides available for hybridization (Brown and Anthony, 2000). We further found that stronger hybridization signals were observed with longer (> 50 bp) probes, which accorded with earlier reports (Letowski *et al*., 2004; Rhee *et al*., 2004; Tiquia *et al*., 2004). Of the 48 oligonucleotide probes we screened, 17 probes with 100% sensitivity to the target DNA and 100% selectivity for the target species were selected for use in microarrays. All of the selected probes were 50 bp or longer in length, with the exception of the 28 bp Phine probe, which was shortened to increase specificity. To cater to the lower melting temperature (T*m*) of this shorter probe, the hybridization temperature of our microarrays was adjusted to 48°C; this did not compromise the specificity of longer probes.

Multiplex PCR‐based methods have previously been used to identify *Phellinus* s.l. at a generic rank (Guglielmo *et al*., 2007; 2008), and ITS sequences have been used in the development of primers for PCR‐based *Phellinus* species identification techniques (Nam *et al*., 2002; Gonthier *et al*., 2015). It is known that microarray techniques can facilitate the simultaneous identification of multiple pathogenic species in a high‐throughput manner (Martin *et al*., 2000; Lévesque, 2001; Lievens and Thomma, 2005; Lievens *et al*., 2005; Tsui *et al*., 2011). To the best of our understanding, this is the first study to employ probes generated from ITS regions in DNA microarrays for the identification and differentiation of *Phellinus* species, and we elected to use reverse dot‐blot hybridization to develop our arrays, as this method has been shown to result in fewer non‐specific cross‐hybridizations (Lévesque *et al*., 1998). We spotted the 17 probes selected for sensitivity and specificity against target *Phellinus* species on to nylon membranes or PVC chips, and further added an amplification control and hybridization control (Fig. 1A). The amplification control was developed from PC1 control DNA, which can be amplified with universal ITS 1/4 primers in the same manner as *Phellinus* target DNA (White *et al*., 1990). To eliminate the possibility of competitive hybridization for amplicons between *Phellinus* probes and the PC1 amplification control probe, the former were designed to target only sense amplicons, while the latter targets only antisense amplicons. An additional hybridization control (HC1) DNA amplicon was also added to the hybridization buffer. Together, these two controls can provide verification of the amplification and hybridization process within our developed assay.

Amplicons derived from template DNA and field samples were labelled with DIG‐dNTP, DIG‐tagged primers, biotin‐dNTP or biotin‐tagged primers. Combined with the use of nylon membrane or PVC chip arrays, this allows for eight different array combinations. Based on the data obtained in this study, the optimal array system utilizes biotin‐tagged primers for labelling, and PVC chips for the array platform. Excluding the time required for sequence amplification in samples, our array system can complete screening and detection in less than 2 h, and is capable of detecting *P. weirii* or *P. noxius* at 1 pg of starting DNA (Fig. 2). The other seven developed systems (Fig. 1B, Fig. S1) display comparable levels of robustness, and together, these results demonstrate the broad applicability of our designed *Phellinus* probes to different array systems.

In this study, we showed that our microarray systems were capable of detecting target *Phellinus* species at about 1 pg of starting DNA (Fig. 2). Considering that the genome of *Phellinus* species is around 40 MB in length, at an average molecular weight of 660 Da for each nucleotide base pair, only 20–30 spores or cells would be needed for detection. However, it is important to note that if non‐target DNA amplified with the same set of primers exceeds target DNA, the detection limit may be affected (Lievens and Thomma, 2005; Lievens *et al*., 2005). We also found that the choice of nylon membrane or PVC chip for the array platform did not noticeably affect detection sensitivity; however, the detection limit for biotin‐labelled amplicons was slightly lower than DIG‐labelled sequences, indicating greater sensitivity with biotin labelling. Since DIG labelling is generally considered to exhibit greater sensitivity than biotin labelling (Rihn *et al*., 1995a,b; Gauthier and Blais, 2003), we believe our findings may be the result of differences in streptavidin‐alkaline phosphatase concentrations, and/or the number of alkaline phosphatases conjugated to each streptavidin molecule.

Our array systems could potentially be used to provide early detection of infestations involving multiple *Phellinus* species, and while general inferences to the disease‐causing organism can be made from the location and species of infected trees (Hansen and Goheen, 2000; Ann *et al*., 2002), such an array could provide much more definitive identification of disease causative agents, thus facilitating the effective implementation of control or quarantine measures. Interestingly, traditional Asian medicine has long considered *P. linteus* to have important medicinal properties, and recent studies have also isolated potentially useful compounds with immuno‐stimulatory or anti‐cancer properties from this species (Nam *et al*., 2002; Dai *et al*., 2010; Wu *et al*., 2012). However, there are major difficulties in differentiating between *P. linteus* and other species, such as *P. igniarius*, *P. laevigatus* and *P. baumii* through phenotypic methods. Considering that *Phellinus* species do not have the same medicinal effects, and that traditional Asian medicine relies on the use of the entire fruiting body or basidiocarp, our microarray system could plausibly be used to provide identification of important medicinal *Phellinus* species, in addition to more conventional applications in disease detection and phytosanitary inspection.

In conclusion, here we describe a set of eight DNA microarray systems that utilize probes generated from ITS regions to simultaneously detect 17 key *Phellinus* species. These arrays were shown to be capable of sensitive, specific, rapid and reliable detection, and could thus provide a significant advantage over traditional morphological or biochemical methods.

## Experimental procedures

### Fungal strains and growth conditions

Cultures of *Phellinus* strains were obtained from the Bioresource Collection and Research Center (BCRC, Hsinchu, Taiwan), the Taiwan Forestry Research Institute (TFRI, Taipei, Taiwan) and the USDA Forest Product Laboratory (USDA FPL, Madison, WI, USA) (Table 1). All strains were grown on potato dextrose agar medium (PDA; BD Difco, Sparks, MD, USA) in the dark at 24°C for 7 days prior to DNA extraction.

### Genomic DNA extraction

Genomic DNA was isolated from fresh fungal cultures, field samples or herbarium specimens, using a modified version of the CTAB method that was previously described (Doyle and Doyle, 1987). In brief, 0.1 g of mycelium or 0.3 g of field or herbarium specimens were placed in 1.5 ml centrifuge tubes containing 500 μl of preheated (65°C) CTAB isolation buffer (2% hexadecyltrimethylammonium bromide, 1.4 M NaCl, 20 mM EDTA, 100 mM Tris‐HCl, pH 8.0) and crushed with a grinder tube, after which 3 μl of 2‐mercaptoethanol was added. The mixture was vortexed for 30 s and then incubated at 65°C for 10 min. The supernatant was extracted with phenol‐chloroform‐isoamyl alcohol (25:24:1, v/v) and centrifuged at 10 000 × *g* for 2 min, after which 0.6 volumes of isopropanol was used to precipitate nucleic acids. The precipitated DNA was washed with 500 μl of wash buffer (76% ethanol, 10 mM ammonium acetate) and re‐suspended in 20 μl of distilled deionized water containing 0.1 μl of RNase A (1 mg/ml concentration; Sigma, St. Louis, MO, USA). Concentration of DNA was determined through spectrophotometry (Nanodrop ND‐1000, NanoDrop Technologies, Rockland, DE, USA).

### DNA amplification and labelling

Universal fungal primers ITS1/4 (White *et al*., 1990) were used to amplify the ITS1–5.8S–ITS2 region in target *Phellinus* species. Amplification was carried out in a 50 μl reaction volume, containing 50 μM of each primer, 10 mM dNTP (GeneTeks BioScience, Taipei, Taiwan), 1 U Prime Taq DNA polymerase (GeNet Bio, Chungnam, South Korea) and 5 μl (∼ 1 ng) of template DNA, using the GeneAmp PCR 2400 System (PerkinElmer, Waltham, MA, USA). Polymerase chain reaction conditions were as follows: 94°C for 4 min and 35 cycles at 95°C for 30 s, 50°C for 30 s and 72°C for 1 min, with a final extension at 72°C for 7 min. Products of PCR were subjected to electrophoresis in 1.0 % (wt/vol) agarose gel and visualized by UV illumination after ethidium bromide staining.

For array analysis, ITS1/4 primers were also used to amplify ITS regions, and the amplicons were simultaneously labelled with either biotin or DIG. Biotin labelling was conducted using either 5'‐biotin‐tagged ITS 1/4 primer sets or biotin‐16‐dUTP mix (Roche Applied Science, Mannheim, Germany), which were added to the amplification reaction. Digoxigenin labelling was performed with either 5'‐DIG‐tagged ITS 1/4 primers or DIG‐11‐dUTP (PCR DIG Labeling Mixplus, Roche Applied Science). Polymerase chain reaction conditions for labelling were the same as described above, and the labelled amplicons were used as targets in subsequent microarray hybridization reactions.

### Oligonucleotide probe design

Species‐specific probes were designed by aligning the ITS sequences of *Phellinus* species, using the AlignX function in Vector NTI (Invitrogen, Carlsbad, CA, USA), to identify polymorphic regions specific to each species. Probes were designed according to the following criteria: (i) probe length of 25–55 bp; (ii) GC% of ∼ 40%; (iii) and T*m* of 55–65°C. The Gibbs free energy (ΔG) of probes was calculated, and the presence of dimers and hairpin loops was assessed in order to minimize the formation of secondary structures. Seven additional thymine bases were added to the 3'‐end of each probe to increase sensitivity (Brown and Anthony, 2000). Conserved regions of the 5.8S rRNA gene and ketosynthase domain were respectively used as positive PCR/hybridization or hybridization‐only controls.

### Oligonucleotide array preparation

Species‐specific probes were synthesized by MDBio (Taipei, Taiwan), and diluted to a final concentration of 20 μM and 10 μM for spotting on positively charged PVC (polyvinylchloride) chips (Dr Chip Biotechnology, Miaoli, Taiwan) or positively charged nylon membranes (Bio‐Rad Laboratories, Hercules, CA, USA) respectively. Oligonucleotide probes were spotted onto PVC chips using automatic spotter, and fixed with UV Crosslinker (Spectrolinker XL‐1000, Spectronics, Westbury, NY, USA). Alternatively, probes were spotted onto nylon membranes using EZspot arrayer (EZlife Technology, Taipei, Taiwan), air‐dried and exposed to UV for probe immobilization.

### Reverse dot‐blot hybridization

Hybridization of amplicons to probes on microarrays was performed according to a previously described protocol (Hsiao *et al*., 2005), with modifications. Briefly, 4 μl of labelled amplicons were added to 220 μl of hybridization solution [5 × SSC (v/v), 2% blocking reagent (w/v; Roche Applied Science), 0.1% N‐lauroylsarcosine (w/v) and 0.02% SDS (w/v)], denatured with boiling water for 7 min, and immediately chilled on ice for 10 min. Hybridization was conducted at 58°C for 40 min, and arrays were then washed twice at 56°C for 5 min each with 0.25 × SSC to remove non‐hybridized PCR products, and incubated for 30 min with 200 μl of blocking solution [1% (wt/vol) blocking reagent dissolved in maleic acid buffer (0.1 M maleic acid, 0.15 M NaCl, pH 7.5)] containing either anti‐DIG‐AP (1:5,000 dilution; Roche Applied Science) or streptavidin‐AP (1:1,000 dilution; Roche Applied Science). Signals were colour developed with 500 μl nitroblue tetrazolium (NBT)/5‐bromo‐6‐chloro‐3‐indolyl phosphate, p‐toluidine salt (BCIP) solution (Roche Applied Science) at room temperature without shaking. PVC chip signals were captured and analysed with Dr AiM reader (Dr Chip Biotechnology), while nylon membrane signals were captured with a BioSpectrum Imaging System (UVP, Upland, CA, USA) and analysed with VisionWorks LS Analysis Software v6.5.2 (UVP).

### Sensitivity analysis

To assess array sensitivity, *P. weirii* and *P. noxius* template genomic DNA was quantified using a NanoDrop ND‐1000 Spectrophotometer (NanoDrop Technologies), diluted to starting DNA concentrations of 1 ng, 100 pg, 10 pg, 1 pg, 100 fg and 10 fg, and subjected to PCR amplification with biotin‐labelled or DIG‐labelled ITS1/4 primers. The PCR products were then subjected to electrophoresis on a 1.0% (w/v) agarose gel, as well as hybridization with PVC chip or nylon membrane microarrays, according to the procedures described above.

### Detection and identification of phellinus species in complex samples and field samples

To ascertain if our array systems were capable of simultaneously identifying multiple *Phellinus* species in a single assay, complex samples containing template genomic DNA from 2 (*P. melleoporus* and *P. noxius*), 3 (*P. melleoporus*, *P. noxius* and *P. pini*) or 4 (*P. melleoporus*, *P. noxius*, *P. pini* and *P. weirii*) *Phellinus* species were prepared, labelled with biotin‐tagged primers during amplification and hybridized to a PVC chip array. In addition, field samples collected from roots, stems, soil and herbariums throughout Taiwan were similarly prepared and hybridized to PVC chip arrays. The field samples were also subjected to conventional detection involving cultivation on PDA or semi‐selective media (Chang, 1995) followed by microscopic examination; and the results were compared with those derived from microarray analysis.

## Conflict of interest

The authors of this article declare no conflicts of interest.

## Supporting information


**Fig. S1.** Reverse hybridization of differentially labelled amplicons to *Phellinus* probes spotted on nylon membrane or PVC chip arrays. Probes were arranged on arrays as indicated in Fig. 1A.
A. Digoxigenin‐deoxynucleoside triphosphate‐labelled amplicons hybridized to probes on nylon membrane.
B. Digoxigenin primer‐labelled amplicons hybridized to probes on nylon membrane.
C. Biotin‐dNTP‐labelled amplicons hybridized to probes on nylon membrane.
D. Digoxigenin‐deoxynucleoside triphosphate‐labelled amplicons hybridized to probes on PVC chip.
E. Biotin‐dNTP‐labelled amplicons hybridized to probes on PVC chip.
F. Biotin‐primer‐labelled amplicons hybridized to probes on PVC chip. For results of biotin‐primer‐labelled amplicons hybridized to probes on nylon membrane, and DIG‐primer‐labelled amplicons hybridized to probes on PVC chip, please see Fig. 1B and C respectively.
**Fig. S2.** Microarray analysis results of field samples collected from trees in Taiwan with suspected or confirmed *Phellinus* infestations. Probes were arranged on arrays as indicated in Fig. 1A. Microarray analysis results for (A) *D. longan*; (B) *C. camphora*; (C) *G. robusta*; (D) *F. microcarpa*; and (E) *P. campanulata* are depicted here.
**Fig. S3.** Microarray analysis results of five species of tree seedlings from a local plant nursery. Probes were arranged on arrays as indicated in Fig. 1A. (A) *F. formosana*; (B) *C. camphora*; (C) *K. elegans*; (D) *M. champaca*; and (E) *A. confusa* seedlings were found to be free of Phellinus infestation through microarray analysis.
