# Supplementary material for: Development of oligonucleotide microarrays for simultaneous multi‐species identification of P hellinus tree‐pathogenic fungi
Source: Microb Biotechnol. 2016 Feb 8;9(2):235–44. doi: 10.1111/1751-7915.12341 (PMC4767280; doi:10.1111/1751-7915.12341)
Supplement: Supplementary file 1 — Fig. S1. Reverse hybridization of differentially labelled amplicons to Phellinus probes spotted on nylon membrane or PVC chip arrays. Probes were arranged on arrays as indicated in Fig. 1A. A. Digoxigenin‐deoxynucleoside triphosphate‐labelled amplicons hybridized to probes on nylon membrane. B. Digoxigenin primer‐labelled amplicons hybridized to probes on nylon membrane. C. Biotin‐dNTP‐labelled amplicons hybridized to probes on nylon membrane. D. Digoxigenin‐deoxynucleoside triphosphate‐labelled amplicons hybridized to probes on PVC chip. E. Biotin‐dNTP‐labelled amplicons hybridized to probes on PVC chip. F. Biotin‐primer‐labelled amplicons hybridized to probes on PVC chip. For results of biotin‐primer‐labelled amplicons hybridized to probes on nylon membrane, and DIG‐primer‐labelled amplicons hybridized to probes on PVC chip, please see Fig. 1B and C respectively. Fig. S2. Microarray analysis results of field samples collected from trees in Taiwan with suspected or confirmed Phellinus infestations. Probes were arranged on arrays as indicated in Fig. 1A. Microarray analysis results for (A) D. longan; (B) C. camphora; (C) G. robusta; (D) F. microcarpa; and (E) P. campanulata are depicted here. Fig. S3. Microarray analysis results of five species of tree seedlings from a local plant nursery. Probes were arranged on arrays as indicated in Fig. 1A. (A) F. formosana; (B) C. camphora; (C) K. elegans; (D) M. champaca; and (E) A. confusa seedlings were found to be free of Phellinus infestation through microarray analysis. [file MBT2-9-235-s001.pdf]

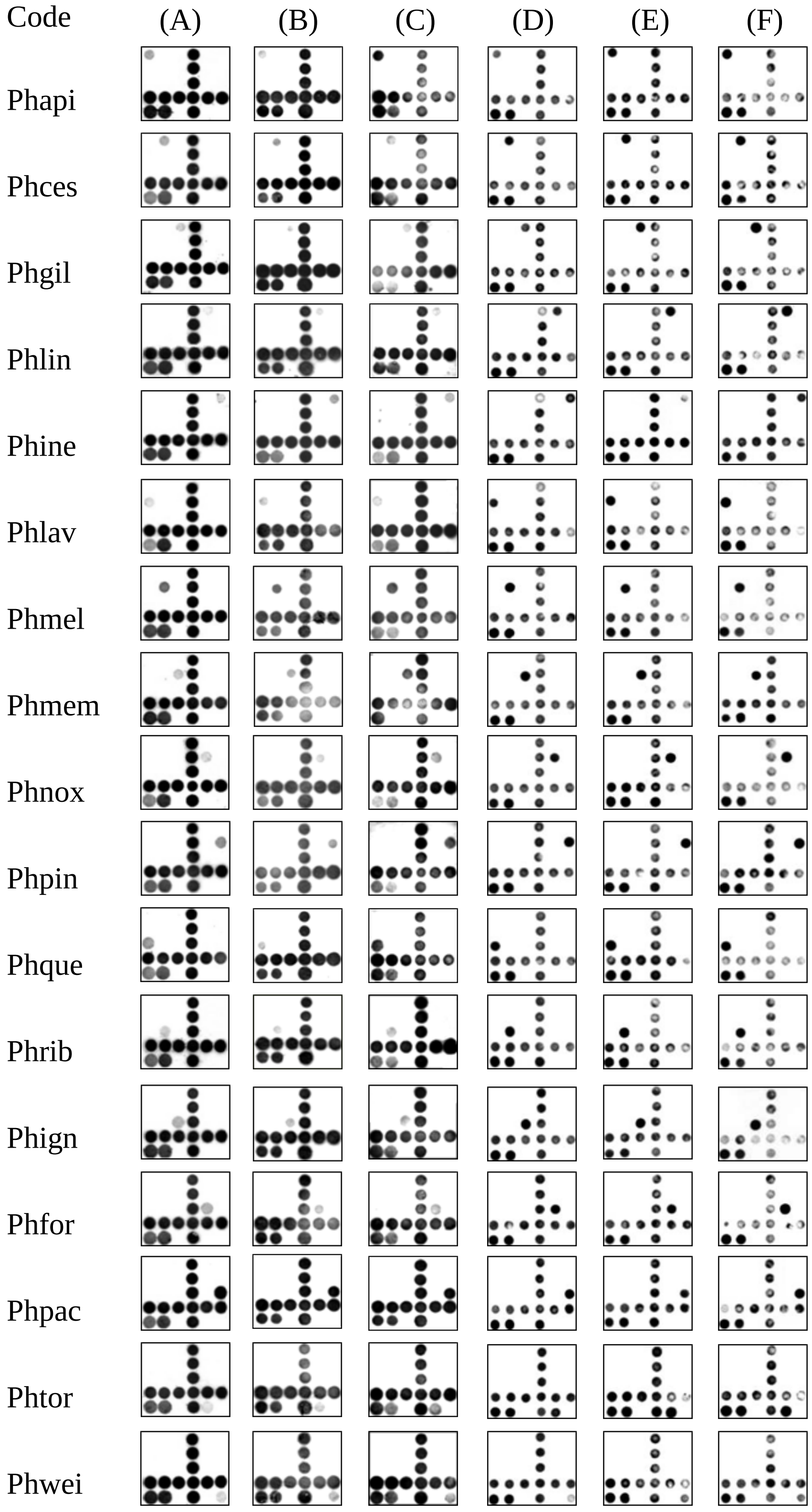

Fig. S1. Reverse hybridization of differentially labeled amplicons to *Phellinus* probes spotted on nylon membrane or PVC chip arrays. Probes were arranged on arrays as indicated in Fig. 1A. (A) DIG-dNTP-labeled amplicons hybridized to probes on nylon membrane. (B) DIG-primer-labeled amplicons hybridized to probes on nylon membrane. (C) Biotin-dNTP-labeled amplicons hybridized to probes on nylon membrane. (D) DIG-dNTP-labeled amplicons hybridized to probes on PVC chip. (E) Biotin-dNTP-labeled amplicons hybridized to probes on PVC chip. (F) Biotin-primer-labeled amplicons hybridized to probes on PVC chip. For results of biotin-primer-labeled amplicons hybridized to probes on nylon membrane, and DIG-primer-labeled amplicons hybridized to probes on PVC chip, please see Fig. 1B and 1C, respectively.

(A) (B) (C) (D) (E)

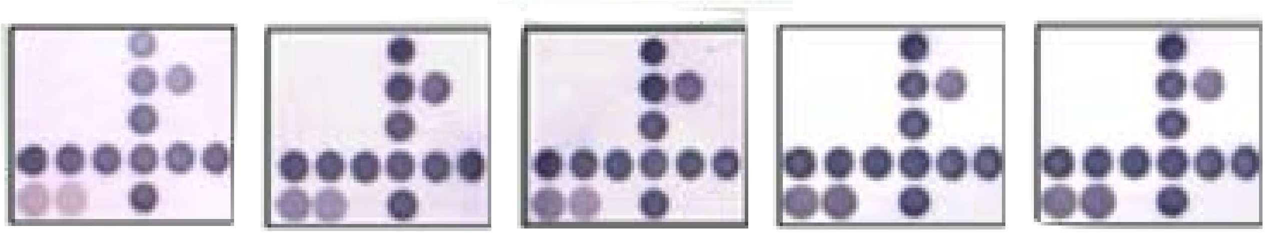

Fig. S2. Microarray analysis results of field samples collected from trees in Taiwan with suspected or confirmed *Phellinus* infestations. Probes were arranged on arrays as indicated in Fig. 1A. Microarray analysis results for (A) *Dimocarpus longan*; (B) *Cinnamomum camphora*; (C) *Grevillea robusta*; (D) *Ficus microcarpa*; and (E) *Prunus campanulata* are depicted here.

(A) (B) (C) (D) (E)

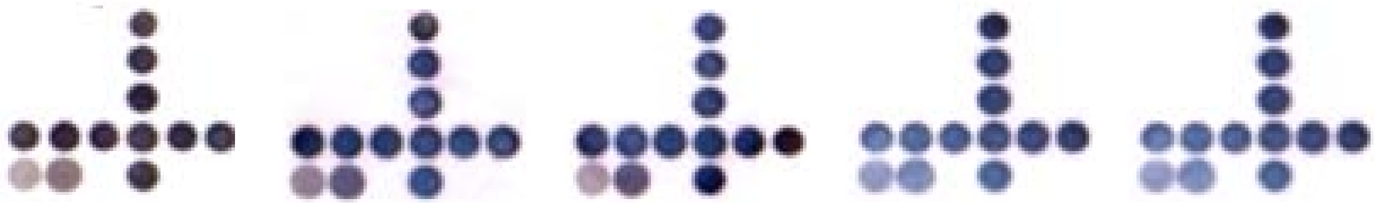

Fig. S3. Microarray analysis results of five species of tree seedlings from a local plant nursery. Probes were arranged on arrays as indicated in Fig. 1A. (A) *Fraxinus formosana*; (B) *Cinnamomum camphora*; (C) *Koelreuteria elegans*; (D) *Michelia champaca*; and (E) *Acacia confusa* seedlings were found to be free of Phellinus infestation through microarray analysis.
